# Buccal Buspirone as add–on Therapy to Omeprazole Versus Omeprazole in Treatment of Gastroesophageal Reflux Diseases (GERD)

**DOI:** 10.22037/ijpr.2020.113320.14231

**Published:** 2020

**Authors:** Saeed Abdi, Zahra Sargashteh, Mohammad Abbasinazari, Jamshid Salamzadeh, Seyed Alireza Mortazavi

**Affiliations:** a *Gastroenterology and Liver Diseases Research Center, Research Institute for Gastroenterology and Liver Diseases, Shahid Beheshti University of Medical Sciences, Tehran, Iran. *; b *Department of Clinical Pharmacy, School of Pharmacy, Shahid Beheshti University of Medical Sciences, Tehran, Iran. *; c *Food Safety Research Center, Shahid Beheshti University of Medical Sciences, Tehran, Iran. *; d *Department of Pharmaceutics, School of Pharmacy, Shahid Beheshti University of Medical Sciences, Tehran, Iran. *

**Keywords:** Buspirone, GERD, Omeprazole, Trial, Buccal

## Abstract

Proton pump inhibitors (PPIs) are recommended as first line treatments for gastroesophageal reflux disease (GERD). Failure to PPIs has been mentioned as a problem in pharmacotherapy of GERD. The present study compared the symptom relief, quality of life (QoL) and adverse drug reactions (ADRs) of omeprazole plus buccal buspirone with that of omeprazole alone.This was a prospective, randomized trial between buccal buspirone (10 mg/d) plus omeprazole (20 mg/d) and omeprazole (20 mg/d) plus placebo administered for 4 weeks to patients with GERD symptoms. Patients who had GERD symptoms enrolled in this study. 67 patients were randomly assigned to either the buspirone plus omeprazole group (n = 33) or the placebo plus omeprazole group (n = 34). Finally, 58 patients completed the study (29 in each group). Treatment response rates in each drug group were evaluated according to the Frequency Scale for the Symptoms of GERD (FFSG). The QoL and ADRs have been also evaluated too.The treatment score rates for symptom relief according to the FFSG were 7.13 ± 5.13 in the buspirone group and 15.34 ± 8.17 in the placebo group. Regarding FFSG score, there is a significant difference between the groups (*p < *0.0001). QoL were 6.86 ± 6.65 and 27.2 ± 20.95 in placebo and buspirone group, respectively after four weeks and there is a significant difference in two groups (* p < *0.0001).The total incidence of ADRs were similar in the buspirone and placebo groups (*p = *0.36).A combination of buccal buspirone plus omeprazole may be a more effective treatment for GERD than omeprazole alone.

## Introduction

Gastroesophageal reflux disease (GERD) is a condition in which gastroesophageal reflux causes either esophageal mucosal break, or annoying symptoms, or both (1). Heartburn and acid regurgitation are considered as the most common symptoms of GERD. Up to 25% of individuals in Western countries experience at least monthly symptoms of heartburn, and 5% report heartburn at least once a day (2). Proton pump inhibitors (PPIs) taken in typical dosages are exceedingly effective inducing symptom remission of GERD in majority of patients (3). Given the prior observation that PPIs can be effective even in the absence of endoscopic evidence of injury,many professional societies like American College of Gastroenterology (ACG) continue to support a trial of empiric therapy for GERD, unless alarm signs for indication of immediate upper endoscopy exist (4). Although, PPIs are currently the most effective treatment for GERD, up to 40% of patients with non-erosive reflux disease (NERD) remain symptomatic on standard regimen, and nearly 10–15% of patients with erosive esophagitis do not achieve full remission after eight weeks of treatment (5). Reflux-related and non reflux-related causes have been mentioned for PPIs failure. Poor compliance, improper dosing time, and rapid metabolism due to polymorphism in *CYP2C19* are examples of reflux- related causes and esophageal motor dysfunction has been identified as a cause of non reflux- related PPIs failure (6). 

Animal studies have shown that serotonin has a putative effect in esophageal motor function (7). In few previous studies, buspirone had a stimulatory effect on esophageal pristaltisis and lower esophageal sphincter (LES) (8, 9). It is suggested that main effects of buspirone on gastrointestinal motility are mediated via 5-HT1A receptors (9). The 5-HT1 agonists elicit an increase in amplitude of esophageal motor waves and of lower esophageal sphincter tone in healthy voulenteers. Potential mechanism of buspirone for the oesophageal effects seen in the previous studies could be via a local action on 5-HT1 receptors to cause contraction of human esophageal smooth muscle (10, 11). Tack *et al.* have evaluated effect of buspirone 10 mg, three times per day,versus placebo in the treatment of functional dyspepsia in a randomized, double-blind, placebo-controlled crossover study. They concluded that a four week treatment with buspirone can be more effective on relieving symptoms of dyspepsia and gastric accommodation compared to placebo (12). In a double blind controlled study, Karamanolis *et al. *investigated the effects of a four weeks buspirone administration on improvement of esophageal motor function in systemic sclerosis patients. 

They have reported that buspirone 20 mg daily could potentially improve esophageal function in all of the patients with sclerosis who report reflux symptoms despite receiving standard treatment by PPIs (13). Due to short half- life and low oral bioavailability, buspirone should be administrated at least twice daily in the treatment of anxiety diseases.This may negatively affect medication adherence of the patients (14). The systemic bioavailbility of daily buccal mucoadhesive tablets of buspirone was studied by Kassem *et al.* They concluded that buccal formulation of buspirone has an increase in bioavailability compared to immediate release tablets (15).

The current study was designed to evaluate the effect of co–administration of buccal buspirone 10 mg/day plus omeprazole 20 mg/day versus omeprazole 20 mg/day plus placebo of buccal buspirone on alleviation of the symptoms of GERD. The primary outcome was the comparison of cure rate of GERD symptoms after four weeks on the basis of a valid questionnaire between the two groups. We also compaired the quality of life, and adverse drug reaction (ADR) incidence between buccal buspirone and placebo groups.

## Experimental

A randomized, double blind placebo-controlled comparative clinical trial was designed and conducted between September 2018 and March 2019 in accordance with the declaration of Helsinki. All participants gave their written informed consent prior to study enrolment. Also the trial was registered in Iranian clinical trial registry site with registration number of IRCT20121021011192N7.

Buccal buspirone and placebo were prepared in pharmaceutical labatory of shahid beheshti university of medical science. *In-vitro* evaluation of buccal tablet consists of physical characterization (content uniformity,weight variation,tickness,hardness and friability), drug release study, mucoadhesion strength measurment, and swelling index study. After acceptance of all mentioned *in-vitro* tests, buccal buspirone and placebo have been prepared for the clinical trial.

The patients more than 18 years old experiencing heartburn and/or regurgitation for two or more days per week were eligible to enter the study. The mentioned symptoms have been repoted in the patients for the first time and they were selected from a well known gastroenterology clinic in Tehran, Iran (Gastroenterology clinic of Taleghani hospital, affiliated to Shahid Beheshti University of Medical Sciences). To confirm the diagnosis of GERD, the Frequency Scale for the Symptoms of GERD (FSSG score) was determined for each patient before enrolment in the study (Primary FSSG). The FSSG questionnaire comprises 12 questions in two domains, reflux symptom, and dysmotility symptom domains. The FSSG uses a 5-point likertscale (never = 0,occasionally = 1, sometimes = 2, often = 3, and always = 4) (16).The exclusion criteria were as follows: serious gastrointestinal disease (gastric or duodenal ulcer), pregnancy, currently using medications that inhibit or neutralize stomach acid, using any medication which affects lower esophageal sphincter (such as nitrates and opioids), hepatic disease (Child–Pugh C), severe renal impairment**,** taking any medications which might interact with buspirone or omeprazole (such as MAO Inhibitors and warfarin) and a history of drug allergy to any of PPIs or buspirone (17, 18).

The patients were randomly allocated to receive omeprazole 20 mg/d plus buccal buspirone 10 mg/d or omeprazole 20 mg/d plus placebo which was identical to buccal buspirone. The patients were allocated randomly in 1:1 ratio in to one of two study groups, intervention or placebo, based on a computer generated random list. To keep participating and clinicians blinded to patient allocation, buspirone tablets and placebo were prepared by the pharmacy of the hospital. At the time of patients enrollment, the pharmacy delivered one tablet box labeled with patient codes depending on their allocation. An online statistical computing web program was used to randomize the participants placement (www.graphpad.com/quickcalcs/randomize1.cfm). The patients in both groups received lifestyle modification recommendation in a written form period. The patients took the study medications daily for four weeks and then returned to the clinic for follow up. The patients were asked to put buspirone or placebo in the buccal area just after breakfast for at least four hours. Also the participants have recommended to swallow omeprazole at around 30 min before breakfast. At the end of four weeks, all of the patients completed FSSGS questionnaire again (Secondary FSSG). The quality of life (QoL) of the patients was also evaluated using a Persian validated questionnaire. This questionnaire consists of 25 items and could be scored between a minimum of 25 (the worst quality of life) to maximum of 175 (the best quality of life) (19). The studied patients were also asked to report any ADR during the trial .Naranjo questionnaire was used for causalty assessment of ADRs in this trial. This is a questionnaire designed by Naranjoet *et al.* for determining the likelihood of whether an ADR is actually due to the medication rather than the result of other factors (20). If the patients had refered to any suspected ADRs during the trial, Naranjo scale was determined and defenite or probable ones were considered as an ADRs. During the study, adherence to medication was evaluated by at least two telephone calls, and it was recorded. Therapy adherence was considered in the patients who reported that they took more than 80% of their tablets (21).

The sample size was estimated based on the statistical analysis of the improvements in the FSSG scores in each group. Previous studies have shown that after four weeks about 50% of the patients with GERD have adequate improvements in their reflux symptoms when they are treated with a standard PPIs dose (22, 23). In this trial, the predicted improvements in the FSSG scores were estimated to be 70% in buccal buspirone group and 50% in placebo group. As a result, a minimum of 27 patients were required in each group to detect a significant difference between the groups with an α value of 0.05 and a power of 0.8. Data were analyzed using the Statistical Package for the Social Sciences (SPSS version 20; IBM Corp., Chicago, Illinois, USA) and p values of less than 0.05 were considered statistically significant**. **Comparisons between buspirone and placebo groups were performed using independent samples *t*-test, Mann–Whitney test, and Pearson’s Chi-squared test as appropriate.

## Results

**Figure 1:**
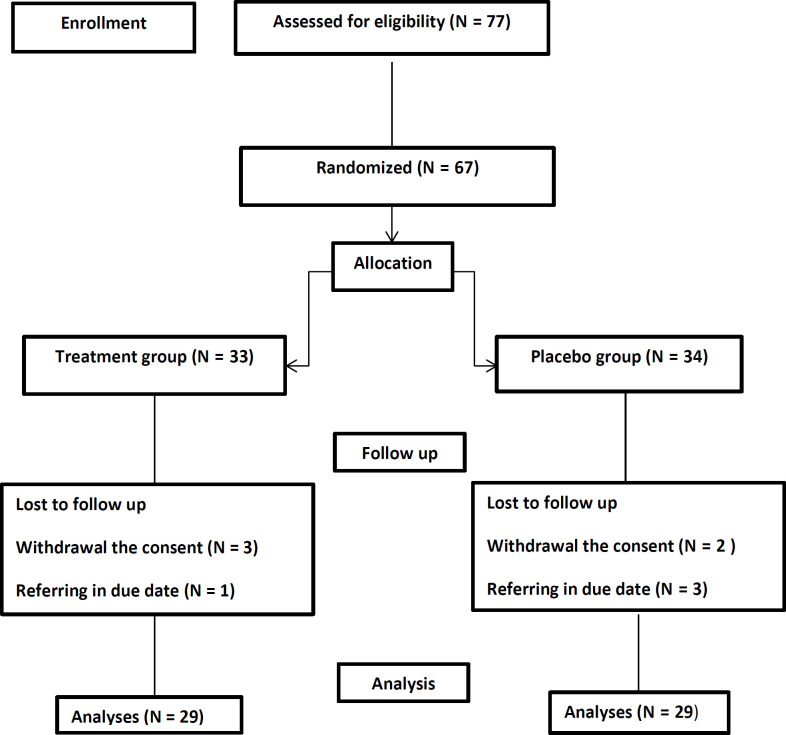
The flow of the study

Among 77 eligible patients enrolled in the study, 19 were excluded and finally, 58 patients completed the study. The ﬂowchart is shown in figure1. The demographic characteristic including mean age,sex, body mass index (BMI), and smoking habit of participants have been shown in Table 1. No significant difference were between study groups regarding sex, BMI, and smoking habits (*p =*0.43*, p = *0.86*, p = *0.74 respectively). Although there was a significantly difference between the groups regarding age distribution of the patients, this could not affect the results of our study considerably (*p *= 0.04). 

In Table 2, FSSG scores of study groups have been shown at the baseline and at the end of the fourth week of study. Comparison of the baseline FSSG scores showed that there was not a significant difference between study groups (*p = *0.67).Within group analyses revealed that after four weeks, FSSG score were significantly reduced in the both (*p < *0.0001) groups; however based on between group analyses, its reduction was greater in the buccal buspirone group. Also, at the end of the fourth week, there was a significance difference in the FSSG score between the two groups (*p *< 0.000). 

In Table 3 the QoL in buspirone and placebo group have been shown after four weeks. QoL was better in buccal buspirone group and analysis showed that there is a significant difference regarding QoL after four weeks in two groups (*p < *0.0001).

In Table 4, incidence rates of ADRs reported by the participants have been shown. The most common ADRs were Headache and vertigo (in six patients). Nevertheless, there was no drop out because of ADRs. Overall, between groups comparison revealed no significant difference in the incidence rates of the ADRs (*p = *0.36). 

## Discussion

Alternative treatments with greater effectiveness and fewer adverse effects are still required for widespread use in the vast majority of GERD patients (6). In recent years buspirone has shown efficacy in treatment of upper gastrointestinal disorders in a limited studies (12, 13). It is documented that Buspirone has a short half life and extensive first pass metabolism. These facts led us to design the study to compare the effects of addition of buccal buspirone to omeprazole versus omeprazole plus a placebo on relief of the early symptoms of GERD. To the best of our knowledge (from searching in medical databases such as SCOPUS, pubmed, and science citation index databases), there is no similar report on this concern.

Since there is an overlap between symptoms of upper gastrointestinal disorders, we used a validated questionnaire (FSSG questionnaire) to assure accurate evaluation of GERD. FSSG score is a useful tool for family practitioners and other health care providers in diagnosing and treating GERD without initial specialist referral or endoscopy (16). Also there is not any difference between the two groups regarding smoking habits. Smith *et al.* have reported that cigarette smoking is known to adversely affect the defence mechanisms against reflux of acid gastric contents into the esophagus and it can be considered as a confounding factor in trials of GERD therapy (24). In our study, there was no significant difference in the gender distribution of the two groups (*p = *0.43*)*, howevere, mean age of the patients in the two groups were significantly different (*p = *0.04). No relationship was shown between prevalence and severity of GERD with sex of the patients. Although, GERD may be prevalent in all age groups, only one study showed that age is associated with GERD. On the other hand,no study has yet shown that male patients are in a higher risk of GERD (25).

Tack *et al. *have done randomized, double–blind, placebo-controlled, and crossover study of 77 patients on the effects of buspirone on functional dyspepsia. Their study included two treatment periods of 4 weeks, separated by a two week washout period. In the first period, the volunteers were given buspirone 10 mg 3 times daily for 4 weeks or placebo 15 min before meals, then the patients switched groups for the second period. They evaluated meal-related symptoms and severity, along with gastric sensitivity, accommodation, and emptying before and after 4 weeks of treatment. They concluded that in the patients with functional dyspepsia, four weeks of administration of buspirone could significantly improve symptoms and gastric accommodation compared with those in the placebo group (12). Although, it seems that the evaluated patients are different in our study and Tack *et al***.** study, in real situatioins, differentioal diagnosis of these two diseases can be quite difficult, as substantial overlap exists epidemiologically, symptomatically and even diagnostically (26). They also reported that there was not a significant difference in the adverse events profile of the patients during the buspirone or placebo administration profile. This was in accordance with our findings.

In an open label trial, Karamanolis *et al.* evaluated effects of immediate release buspirone 20 mg/d on esophageal motor function and symptoms in patients with esophageal involvement associated with systemic sclerosis. They have concluded that a 4 week administration of buspirone can improve heartburn and regurgitation scores of the patients *(p = *0.001*, *and* p = *0.022*,* respectively). They evaluated symptoms of the patients such as heartburn, regurgitation, chest pain, and dysphagia by a visual analogue scale. Not being a randomized, placebo–controlled trial and lack of a control arm was an important limitation of the Karamanolis *et al.* study.They used high resolution manometry and chest computed tomography to assess motor function and esophageal dilatation, respectively (13). Jaipal *et al.* have formulated and examined buccal buspirone 10 mg both *in-vitro *and *in-vivo*. They have reported a good profile of bioavailability and safety of buccal buspirone in this dose (27). As there is not any published article regarding safety and efficacy of buccal buspirone in GERD patitents, we decided to choose 10 mg as buspirone dose in our trial.Our limitation was using manometry for assessment of motor function and we recommend doing it in future studies. As it seems that buspirone could improve esophagus preistaltism, evaluation of buccal buspirone on manometric parametres in future studies is important. 

Patient’s adherence is a common challenge in medicinal treatment of disease. It has been shown that single daily use of medications increase adherence significantly (28). In order to examin efficacy of sustained release form of medications on GERD therapy, Abbasinazari *et al.* have evaluated effect of sustained released (SR) baclofen plus omeprazole versus omeprazole alone to control GERD symptoms in a randomized clinical trial. SR baclofen could be administered once daily instead of immediate released baclofen which must be administer three times per day. They have been reported that a combination of SR baclofen and omeprazole may be a more effective treatment for heartburn and regurgitation than omeprazole alone (29). In our trial, we decided to use buccal buspirone instead of immediate release form as it can be used once daily. All patients were adherent to the buccal administration of buspirone and placebo, and no patient was withdrawn from the study because of a difficulty in the administration rout or mucosal side effects of the buccal form.

Our results support the use of buccal buspirone as add–on therapy to omeprazole in patients with GERD. Although, we did not performed esophageal manometry in study subjects, it is recommended this test to be included in future researches. 

**Table 1:** Demographic characteristics of the patients in buccal buspirone and placebo group(N = 58).

		**Buccal buspirone (n = 29)**	**Placebo (n = 29)**	***p*** ***-*** ***value***
Mean age (year) (± SD)		36.27 ± 11.48	42.34 ± 10.87	0.04
Sex	Female	15	18	0.43
	Male	14	11	
Body Mass Index (kg/m^2^) (± SD)		22.7 ± 3.2	22.4 ± 3.1	0.86
Smoker	No	4	6	0.74
	Yes	25	23	

**Table 2:** FFSG^a^ scores in buspirone and placebo group at baseline and after four weeks

**Parameter**	**BuccalBuspirone group(n = 29) (Mean ± SD** ^b^ **)**	**Placebo group (n = 29) (Mean ± SD** ^b^ **)**	***p*** **-value**
Baseline FFSG (BF)	22.51 ± 7.23	23.37 ± 8.13	0.672
Second FSSG (SF)	7.13 ±5.13	15.34 ±8.17	< 0.0001
***p*** **-value**	0.0001	0.0001	

^a^FFSG, Frequency Scale for the Symptoms of GERD.

^b^SD, standard deviation**.**

**Table 3:** Quality of life (QoL) in buccal buspirone and placebo group after four weeks

	**Buccal buspirone group (n = 29)**	**Placebo group (n = 29)**	**P**
Quality of life (± SD)	27.2 ± 20.2	6.86 ± 6.65	< 0.0001

**Table 4:** Incidence of adverse drug reactions in Buccal Buspirone and placebo groups

**Adverse effects**	**Buccal Buspirone (n = 29)**	**Placebo (n = 29)**	**Total**
Headache and vertigo	2	4	6
Stomach pain	2	2	4
Buccal ulcer	0	3	3
Nausea and vomiting	1	1	2
Mouth dryness	0	1	1
